# Willingness to accept human papilloma virus vaccination and its associated factors among parents with eligible daughters in Addis Zemen town, Northwest Ethiopia

**DOI:** 10.1186/s13027-023-00551-6

**Published:** 2023-12-21

**Authors:** Mulugeta Wassie, Alebachew Ferede Zegeye, Wondesen Worku, Tiruye Sisay, Tsadik Eyob, Daniel Ayelegne Gebeyehu

**Affiliations:** 1 School of nursing, College of Medicine and health sciences, University of Gondar, Gondar, Ethiopia; 2 Department of medical nursing, school of nursing, University of Gondar, Gondar, Ethiopia; 3 Department of Psychiatry, school of Medicine, College of Medicine and health sciences, University of Gondar, Gondar, Ethiopia

**Keywords:** HPV vaccine, Willingness, Eligible parents, Daughter

## Abstract

**Background:**

Cervical cancer is one of the most common cancers in women. Evidences show that, routine immunization of girls at age 14 year and immunization of girls at age 9 year through a 5 years extended interval between doses are the most efficient to control the disease. Despite this, there is very little information on parents’ willingness to accept the human papilloma virus vaccine. Therefore, assessing willingness to accept human papilloma virus vaccination and its associated factors among parents with eligible daughter will help to designing, implementing and monitoring effectiveness of HPV vaccine immunization program.

**Methods:**

A community-based cross-sectional study was conducted among 386 parents with eligible daughters from 8July–6August, 2022. The multistage sampling technique was used. Data was collected using an interviewer-administered questionnaire. Responses were coded and entered into the computer using EPI data version 4.606 statistical packages, and SPSS version 23 was used for data analysis. Frequencies, percentages and means were as to describe the study variables in relation to the participants. Bivariable and multivariable logistic regression were employed. The statistical significance was set at a p-value of < 0.05 with its respected odds ratio.

**Results:**

A total of 386 study participants were included in the study. Among participants, 80.3% (95% CI: 76.3, 84) were willing to vaccinate their daughters for HPV vaccination. The parents’ willingness was affected by the male parents ([AOR = 3.5; 95% CI (1.673–7.371)], fear of side effects [AOR = 0.385; 95% CI (0.206–0.718)], and with poor awareness on the HPV vaccine [AOR = 0.483; 95% CI (0.259- 0.900)].

**Conclusion:**

The study has shown that willingness to accept the HPV vaccine is about 80% and significantly affected with parental sex, information on the HPV vaccine, and fear of side effects. As such, it may be helpful for the health care providers and the health care policy makers to emphasize on providing easily understandable information using mass media and social campaign. In addition giving trainings more targeted to female parents might be important.

## Introduction

The projected incidence of cervical cancer(CC) is 13.1 per 100,000 women worldwide but varied extensively in different regions of the globe, with less than 2 per 100,000 women in economically advanced nations to 75/100,000 in low income developing nations [1]. It is the main cause of cancer related mortalities among women in Africa [1].

In the absence of vaccination against human papilloma virus (HPV), 11.6 million cases of CC are expected by 2094 in the globe [2]. About 75% of the disease burden is concentrated in 25 countries mostly found in Africa and Asia [2]. Global immunization with HPV vaccine targeted to HPV types 16 and 18, with cross-protection against HPV types 31, 33, and 45, might prevent about 8.7 million cases [2, 3].

There are more than 100 kinds of HPV types of which at least 14 are cancer-causing.

HPV is mainly transmitted through sexual contact and most people are infected with shortly after the onset of sexual activity. Two HPV types (16 and 18) cause about 70% of cervical cancers and pre-cancerous cervical lesions causing squamous intraepithelial lesions [4–6].

The two effective vaccines composed of HPV strains 16 and 18 are primary prevention strategies of HPV which is the main cause of CC [7]. Strategies involving vaccinating girls aged 9–14 with two doses are predicted to be the most cost-effective in low- and middle-income countries [8]. Evidences showed that, routine immunization of girls at age 14 year and immunization of girls at age 9 year through a 5 years extended interval between doses and a catch up programme at age 14 years are the most efficient to control this cancer [9, 10].

Despite this fact, there is no information regarding the HPV vaccine acceptance and associated factors among parents with eligible daughters in the study setting. Therefore, the aim of this study was to assess willingness to accept human papilloma virus vaccine and its associated factors among parents with eligible daughter in Addis Zemen town, Northwest Ethiopia.

## Methods and materials

### Study design, period and area

A community-based cross-sectional study was conducted from July 8–August 6, 2022. This study was conducted in Addis Zemen town which is located in the Northwestern part of Ethiopia. It is around 637 km away from Addis Ababa, the capital city of Ethiopia and 82 km from Bahir Dar city. According to the current statistics, the town has 4 kebeles and a total of 198,374 population [11].

### Populations

The source population for this study was all parents who had eligible daughter living in Addis Zemen town and the study population was all parents who had eligible daughter in selected kebele at Addis Zemen during the study period. Parents with psychiatric disorder and severely ill during the study period were excluded in the study.

### Sample size determination

The sample size was calculated by using single population proportion formula considering 81.3% vaccine acceptance [12], 95% confidence interval (CI), 5% margin of error (d) and 1.5 design effect:

n = (Z_α/2_)^2^p (1-P)

d^2^

Where, n = initial sample size

Z = 1.96 the corresponding Z-score for the 95% CI

P = Proportion = 81.3%

W = Margin of error = 5%= 0.05

n= $$\frac{\left(1.96\right)2 \text{x} 0.813(1-0.813)}{\left(0.05\right)2}$$= 233.6≈234

This sample size multiplied by design effect of 1.5 and adding 10% non-response rate gives 386.

### Sampling technique and procedure

The multistage sampling method was used. There are four kebeles, and two kebeles were selected by a simple random method and from the selected kebeles, 386 participants were selected by an identification number that was given after a house-to-house visit. Then, a proportional-to-size allocation technique was employed to determine the study participants from each kebele. Finally, the study participants were selected by a simple random sampling method.

### Operational definitions

**Eligible daughter**- young daughters aged 9-14years.

**Knowledge**- There were 16 knowledge questions and every questions have yes, no and I don’t know answer and if the study participant answered 0–4 ‘yes’ out of 16 items was considered as “poor knowledge”, 5–12 “moderate Knowledge”, and 13 − 16 “good knowledge” [12].

**Attitude**-There were 10 questions. Every questions have yes/no answer and if the study participant answered 0–5 ‘yes’ out of 10 items was considered as having negative and 6–10 positive attitude [13].

**Willingness to accept the vaccine** – using the question, “Are you willing to vaccinate your daughter for HPV vaccination that can protect against HPV infection?“(options: Yes/No). Those who answered “yes” were considered are willing to accept HPV vaccine and those who answered “no” were considered as they aren’t willing to accept HPV vaccine [12].

### Data collection tools and procedures

Data were collected using structured interviewer administrated questionnaire adapted from the literature [12]. The face-to-face interview method was used to collect all relevant information from respondents. Three nurses with BSc degree were involved in data collection process. For the case if more than one parents were available in the household, data collectors has selected one parent using a lottery method to make interview.

### Data quality control

The questionnaire was prepared in English and translated to the local language (Amharic) by language experts, and back-translated to English to check for consistency of the questionnaire. Before the actual data collection, pre-test was conducted among 5% of the sample size in kebeles other than selected kebeles to evaluate the clarity of questions and validity of the instrument and reaction of respondents to the questions. Every day, all questionnaires were reviewed and checked at the end of the data collection period, and any errors were corrected accordingly with the supervisor and data collectors. Two nurses with MSc educational level supervised the data collection process.

### Data processing and analyzing

After data collection, each questionnaire was checked for completeness and consistency. Responses were coded and entered into the computer using EPI data version 4.606 statistical packages, and SPSS version 23 was used for data analysis. Descriptive statistics like frequencies, percentages and mean were used to describe the study participants in relation to the study variables. Binary logistic regression model was used to identify factors associated with the outcome variable. Variables at a p value < 0.2 in the bivariable analysis were included in the multivariable analysis. Explanatory Variables with p-value < 0.05 and with their respected odds ratio in multivariable analysis were declared as statistically significant.

## Results

### Socio-demographic characteristics

A total of 386 participants were included in this study with a response rate of 100%. The mean age of the respondents was 44.55 years and 140 (36.3%) of the participants were found in the age group between 30 and 39 years and 251 (65%) of respondents were female (Table 1).

**Table 1:** Socio demographic characteristics of parents of daughters in Addis Zemen, North West Ethiopia (n = 386)

Variable (N = 386)	Categories	Frequency(N)	Percent (%)
Sex	Male Female	135 251	35.0 65.0
Age	21–29 30–39 40–49 $\ge$50	3 140 128 115	0.8 36.3 33.2 29.8
Income	Paid Unpaid	114 272	29.5 70.5
Educational status	Can’t read and write only read and write primary school secondary school diploma degree and above	77 86 44 57 64 58	19.9 22.3 11.4 14.8 16.6 15.0
Occupation	civil servant self employed merchant farmer house wife others*	97 83 60 54 70 22	25.1 21.5 15.5 14.0 18.1 5.7

Other* – Daily laborer, retired

### Knowledge towards cervical cancer

One hundred eight six (48.2%) study participants had poor knowledge, 160(41.5%) had moderate knowledge, and 40 (10.1%) had good knowledge towards cervical cancer (Table 2).

**Table 2:** Knowledge towards cervical cancer among parents of eligible daughters in Addis Zemen town, Northwest Ethiopia (n = 386)

Knowledge questions		Responses	
	Yes	No	I don’t know
HPV can cause cervical cancer	155(40.2%)	31(8%)	200(51.8%)
HPV infections are preventable	171(44.3%)	29(7.5%)	186(48.2%)
HPV is sexual transmitted disease	138(35.8%)	50(13.0%)	198(51.3%)
Condom use can prevent HPV infection	105(27.2%)	61(15.8%)	220(57.0%)
HPV last for years	134(34.7%)	27(7.0%)	225(58.3%)
Cervical cancer is caused by persistent HPV infection?	143(37.0%)	25(6.5%)	218(56.5%)
HPV may infect both men and women	108(28.0%)	98(25.4%)	180(46.6%)
Most HPV infection resolves spontaneously	45(11.7%)	136(35.2%)	205(53.1%)
HPV can infect you without symptoms	108(28.0%)	70(18.1%)	208(53.9%)
HPV can cause genital ulcer	165(42.7%)	28(7.3%)	193(50.0%)
HPV can cause other anogenital cancer	108(28.0%)	46(11.9%)	232(60.1%)
HPV vaccine prevents around 70% cervical cancer	142(36.8%)	23(6.0%)	221(57.3%)
Pap-smear can screen cervical cancer.	200(51.8%)	14(3.6%)	172(44.6%)
Pap-smear is very or relatively effective in screening cervical cancer	180(46.6%)	25 (6.5%)	181(46.9%)
Pap-smear should be done every 3 years	117(30.3%)	27(7.0%)	242(62.7%)
Pap-smear can be done the age of 35 and above	148(38.3%)	30(7.8%)	208(53.9%)

### Attitude towards HPV vaccine

From the study participants, 289 (74.9%) had a positive attitude, and the rest, 97 (25.1%), had a negative attitude (Table 3).

**Table 3:** Attitude towards HPV vaccine among parents of daughters in Addis Zemen town, North West Ethiopia (n = 386)

Attitude questions	Responses	
	Yes	No
Do you think cervical cancer is a sever disease	365(94.6%)	21(5.4%)
Do you think cervical cancer is preventable disease	251(65.0%)	135(35.0%)
Do you think your daughter is susceptible to HPV infection	155(40.2%)	231(59.8%)
Do you think HPV vaccine is helpful to prevent cervical cancer?	271(70.2%)	115(29.8%)
Do you think HPV vaccine is safe?	245(63.5%)	141(36.5%)
There is less risk involved in being vaccinated than in having HPV infection?	260(67.4%)	126(32.5%)
HPV vaccine will not lead to complicated sexual activities	212(54.9%)	174(45.1%)
I would not want my children to be infected with HPV	314(8.3%)	72(18.7%)
I would have my children vaccinated against HPV if the vaccination is freely available.	275(71.2%)	111(28.8%)
Information on HPV helps me to decide whether my children should be vaccinated against HPV	299(77.5%)	87(22.5%)

### Information about HPV vaccine and related characteristics

**Fig. 1:**
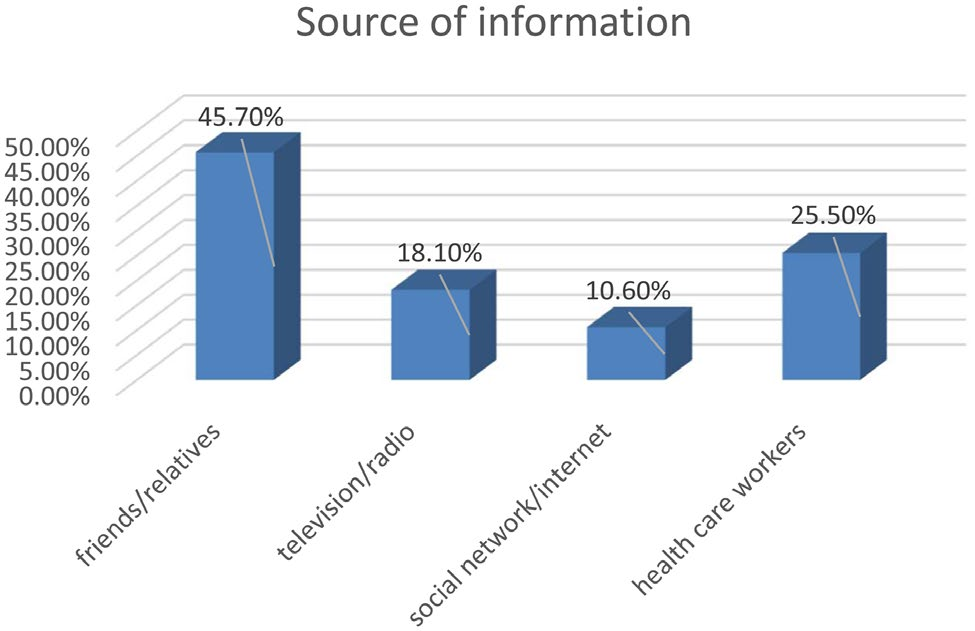
Source of information about HPV vaccine among parents of eligible daughters in Addis Zemen town, Northwest Ethiopia (n = 386). Willingness to Accept HPV vaccine

Among study participants, 302 (78.2%) heard about the HPV vaccine, 202 (52.3%) feared the vaccine’s side effects for their daughters, and almost all participants think the vaccine price is zero (94.8%)(Fig. 1).

### Willingness to accept HPV vaccine

**Fig. 2:**
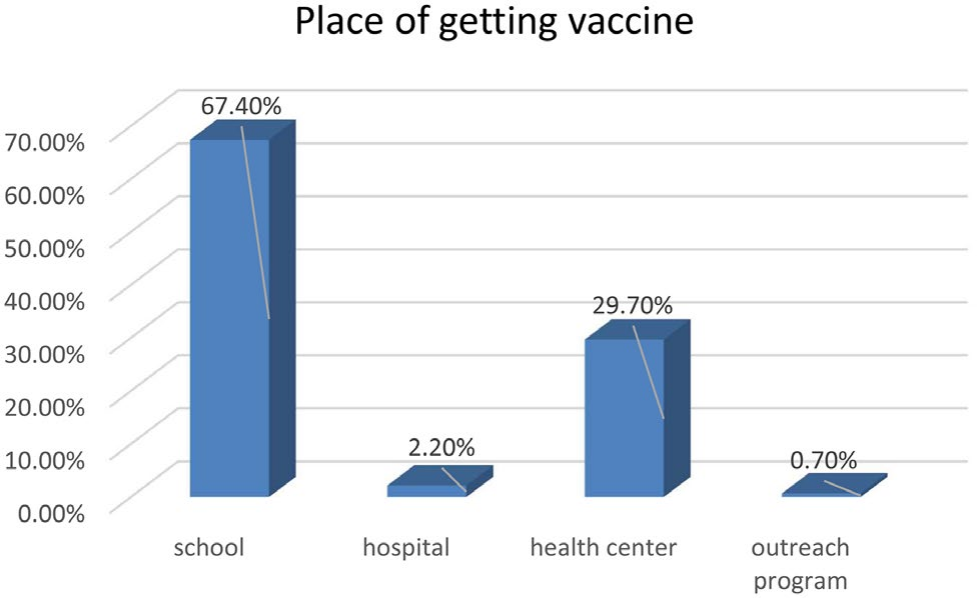
Place of getting HPV vaccine among eligible daughters in Addis Zemen town, Northwest Ethiopia, 2022 (n = 386)

From the total study participants, 80.3%(95%CI:76.3–84) were willing to accept the HPV vaccine for their daughters that can protect HPV infection, and 64.8% of the participants’ daughters got the vaccine before and of which the majority got it in school (Fig. 2).

### Factor associated with willingness to accept HPV vaccine

Binary logistic regression analysis model was employed to identify factors that affect the willingness to accept HPV vaccine. In bivariable analysis, sex, income, educational status, occupation, fear of side effects, and information on the HPV vaccine were associated to the outcome variable at p-value < 0.2. Then, sex, fear of side effects and information on the HPV vaccine were significantly associated to vaccine acceptance at p-value < 0.05 in multivariable analysis.

The study revealed that male parents were about 3.5 times more likely to accept HPV vaccine than female parents [AOR = 3.51; 95% CI (1.673–7.371)].

The study also showed that parents who had a fear of side effects of the HPV vaccine were about 61.5% less likely to accept the vaccine as compared to those who had no fear of side effects [AOR = 0.385; 95% CI (0.206–0.718)].

Parents who had no information on the HPV vaccine were 51.7% less likely to accept the HPV vaccine for their daughters than those who had information [AOR = 0.48; 95% CI (0.25–0.90)] (Table 4).

**Table 4:** Bivariate and Multivariate analysis of factors associated with willingness to accept cervical cancer vaccination among parents of daughters in Addis Zemen town, Northwest, 2022 (n = 386)

Variable	Categories	Willingness to accept		COR (95%CI)	AOR (95%CI)	P -value
		Yes (%)	No (%)			
Sex	Male	94(69.6%)	41(30.4%)	2.69(1.61–4.49)	**3.51(1.67–7.37)**	0.01**
	Female	216(86.1%)	35(13.9%)	1	1	
Income	Paid	105(92.1%)	9(7.9%)	1	1	
	Unpaid	205(75.4%)	67(24.6%)	3.813(1.83–7.94)	2.19(0.48–9.99)	0.31
Educational stats	can’t read and write	48(62.3%)	29(37.7%)	16.91(3.83–74.60)	18.236(2.75–121.00)	0.056
	only read and write	58(67.4%)	28(32.4%)	13.51(3.05–59.42)	14.534(2.32-90.851)	0.07
	primary school	37(84.1%)	7(5.9%)	5.297(1.043–26.91)	3.97(0.55–28.78)	0.172
	secondary school	50(87.7%)	7(12.3%)	3.92(0.77–19.75)	4.58(0.77–27.08)	0.093
	Diploma	61(95.3%)	3(4.7%)	1.37(0.22–8.54)	1.62(0.25–10.59)	0.613
	Degree and above	56(96.6%)	2(3.4%)	1	1	
Occupation	civil servant	89(91.8%)	8(8.2%)	1	1	
	self employed	72(86.7%)	11(13.3%)	1.69(0.649–4.449)	0.248(0.39–1.573)	0.139
	merchant	53(88.3%)	7(11.3%)	1.46(0.50–4.26)	0.15(0.02–1.04)	0.056
	Farmer	31(57.4%)	23(42.6%)	8.25(3.34–20.35)	0.35(0.05–2.42)	0.292
	House wife	51(72.9%)	19(27.1%)	4.14(1.69–10.14)	0.37(0.05–2.65)	0.372
	Others	14(63.6%)	8(36.4%)	6.35(2.03–19.68)	0.91(0.11–7.26)	0.936
Fear of side effect	Yes	178(88.1%)	24(11.9%)	0.34(0.20–0.58)	**0.38(0.21–0.72)**	0.03*
	No	132(71.7%)	52(28.3%)	1	1	
Information on HPV vaccine	Yes	62(66%)	32(34%)	1	1	
	No	248(84.9%)	44(15.1%)	0.34(0.20–0.58)	**0.48(0.25–0.90)**	0.022*

Key p* - $$<$$0.05 P**- $$\le$$0.01

## Discussion

The study was conducted to assess the willingness to accept HPV vaccine and associated factors among parents with eligible daughters in Addis Zemen town.

The study showed that more than 80% of the study participants were willing to accept the HPV vaccine to be given for their eligible daughters. This finding is aligned with studies done in Gondar (81.3%) [14] and Nigeria (81.8%) [15], south Africa (80%) [16] and Denmark (80%) [17]. However, several studies conducted in different study settings such as Addis Ababa (94.3%) [18], Kenya (89%) [19], Tanzania (93%) [20], Honduras (91%) [21] and Malaysia (87.1%) [22] Shows higher acceptance level than the current finding. This discrepancy may be due to socio cultural, literacy level, study setting and health policy differences of the respected countries.

This finding also revealed that the sex of the parents was statistically associated with willingness to accept the vaccine. The current finding shows that males are more likely to show willingness to accept the HPV vaccine, unlike other studies reveal as females show greater willingness than males, like studies conducted in Spain [23] and European countries [24]. This may be due to males are more exposed to the information related to the vaccine in different public meeting and social media than females in the current study area. This is as males are more expected to engage in the outdoor activities but on the contrary females according to the community’s socio cultural context in the study setting and the region at all.

The current research finding revealed that fear of side effects made study participant less likely to accept the HPV vaccine. This finding is aligned with studies done in Hong Kong [25], and China [26]. People prefer not to get vaccines for a variety of reasons, but the main ones are a lack of trust in vaccine safety and fears about side effects [27].

Additionally, this study revealed that information on HPV vaccination was significantly associated with willingness to accept the vaccine. This finding was consistent with a study done in Gondar [12] and Addis Ababa [18]. This might be due to confusion or a lack of information, which are common factors for vaccine hesitancy [28]. Parents who received information about the HPV vaccine may know more about the vaccine property, where it is given, and who gets vaccinated. This might helped informed parents to be willing to accept the HPV vaccine for their daughters.

## Conclusion

The study has shown that willingness to accept the HPV vaccine is about 80% and it was significantly affected with parental sex, information on the HPV vaccine, and fear of side effects. It may be helpful for the health care providers and the health care policy makers to emphasize on providing easily understandable information related to the vaccine using mass media and social campaign. Furthermore, the HPV acceptance rate will be increased if information is disseminated for female parents as well.

### Limitations of the study

This study is cross sectional in nature, so it shares the limitations of cross sectional study design. In addition, there may be social desirability bias which may inflate the proportion of HPV acceptance rate.

## Data Availability

Data will be available upon request from the corresponding author.

## Abbreviations

AOR: Adjusted Odd Ratio
CDC: Centers for Disease Control and Prevention
CI: Confidence Interval
COR: Crude Odd Ratio
HPV: Human Papilloma virus
SPSS: Statistical Package for the Social Sciences
